# Differentiation of parenteral anticoagulants in the prevention and treatment of venous thromboembolism

**DOI:** 10.1186/1477-9560-9-5

**Published:** 2011-03-28

**Authors:** Jawed Fareed, Cafer Adiguzel, Indermohan Thethi

**Affiliations:** 1 Departments of Pathology, Loyola University Medical Center, Maywood, Illinois, USA; 2 Department of Pharmacology, Loyola University Medical Center, Maywood, Illinois, USA; 3 Department of Medicine, Division of Hematology, Marmara University Medical School, Istanbul, Turkey; 4 Department of Medicine, Aurora Memorial Hospital, Burlington, WI, USA

## Abstract

**Background:**

The prevention of venous thromboembolism has been identified as a leading priority in hospital safety. Recommended parenteral anticoagulant agents with different indications for the prevention and treatment of venous thromboembolism include unfractionated heparin, low-molecular-weight heparins and fondaparinux. Prescribing decisions in venous thromboembolism management may seem complex due to the large range of clinical indications and patient types, and the range of anticoagulants available.

**Methods:**

MEDLINE and EMBASE databases were searched to identify relevant original articles.

**Results:**

Low-molecular-weight heparins have nearly replaced unfractionated heparin as the gold standard antithrombotic agent. Low-molecular-weight heparins currently available in the US are enoxaparin, dalteparin, and tinzaparin. Each low-molecular-weight heparin is a distinct pharmacological entity with different licensed indications and available clinical evidence. Enoxaparin is the only low-molecular-weight heparin that is licensed for both venous thromboembolism prophylaxis and treatment. Enoxaparin also has the largest body of clinical evidence supporting its use across the spectrum of venous thromboembolism management and has been used as the reference standard comparator anticoagulant in trials of new anticoagulants. As well as novel oral anticoagulant agents, biosimilar and/or generic low-molecular-weight heparins are now commercially available. Despite similar anticoagulant properties, studies report differences between the branded and biosimilar and/or generic agents and further clinical studies are required to support the use of biosimilar low-molecular-weight heparins. The newer parenteral anticoagulant, fondaparinux, is now also licensed for venous thromboembolism prophylaxis in surgical patients and the treatment of acute deep-vein thrombosis; clinical experience with this anticoagulant is expanding.

**Conclusions:**

Parenteral anticoagulants should be prescribed in accordance with recommended dose regimens for each clinical indication, based on the available clinical evidence for each agent to assure optimal safety and efficacy.

## Introduction

Venous thromboembolism (VTE) is a common disease that occurs in hospitalized surgical and medical patients and in the community [1]. In 2003, over 12 million medical and surgical patients in the Nationwide Inpatient Sample, comprising 31% of all US hospital discharges for that year, were at risk of VTE and warranted thromboprophylaxis [2]. The risk of VTE can persist for a considerable period of time after the VTE-triggering event, such as surgery, or hospitalization for an acute medical condition [3]. The period of increased thrombotic risk may be sustained by the presence of ongoing risk factors such as malignancy or immobility [1]. In addition, the risk of recurrent VTE is high, with nearly one-third of patients experiencing a recurrent event within 8 years [4]. In patients who do suffer a recurrent VTE requiring rehospitalization, 50% of these events occur in the first 3 months after their initial deep-vein thrombosis (DVT) or pulmonary embolism (PE) [5]. After a VTE event, upto one-third of patients may suffer from the post-thrombotic syndrome, which causes long-term morbidity due to leg pain, swelling, and the effects of vascular insufficiency [4].

Effective prevention of VTE has therefore been identified by the Agency for Healthcare Research and Quality as the leading priority in hospital safety practices. Hospitals have the potential to reduce the clinical and economic burden of VTE by implementing hospital-wide protocols for the prevention and treatment of VTE. Several guidelines are available such as those regularly updated by the American College of Chest Physicians (ACCP) and the International Union of Angiology (IUA) [6-8], as well as specialty-based VTE guidelines.

A number of parenteral antithrombotic regimens are available and recommended for the prevention and treatment of VTE, including unfractionated heparin (UFH), low-molecular-weight heparins (LMWHs; enoxaparin, dalteparin, tinzaparin), and selective anti-Xa inhibitors (fondaparinux), as well as oral vitamin K antagonists (warfarin). Due to the number and complexity of indications in VTE management, the choice of antithrombotic agent can appear complicated. In particular, differentiating between the parenteral antithrombotics can be confusing as expert guidelines on VTE usually recommend one of a number of options and 'a LMWH' rather than specifying which LMWH to prescribe. However, LMWHs are distinct pharmacological agents and not clinically interchangeable, as stated by regulatory authorities including the US Food and Drug Administration (FDA), European Medicines Agency (EMEA), World Health Organization, ACCP, American Heart Association, and American College of Cardiology [6,7,9-12]. Therefore, when prescribing a LMWH for either the prevention or treatment of VTE, the clinical evidence for each agent must be reviewed. This review aims to assist this decision-making process by analyzing points of differentiation between each of the parenteral antithrombotic agents recommended in current VTE management guidelines.

## Currently available parenteral anticoagulants for VTE prevention and treatment

UFH has long been used as an anticoagulant in the prevention and treatment of VTE. Prophylaxis with anticoagulants is effective in reducing the incidence of VTE and in treating acute VTE [6,8,13], but is inherently associated with a risk of bleeding complications. UFH use is also limited by the need for regular coagulation monitoring. Over the last 10 years, UFH has been replaced as the reference standard anticoagulant in VTE management by the LMWHs. Three LMWHs are currently available in the USA: enoxaparin, dalteparin, and tinzaparin. These LMWHs are individual pharmacological entities and have different FDA-licensed indications and dosing regimens. More recently, the synthetic pentasaccharide fondaparinux has been developed and FDA approved.

### Mechanism of action

Although UFH, LMWHs, and fondaparinux are all indirect anticoagulants and exert their effects through binding to the plasma cofactor antithrombin (AT) and releasing endogenous mediators, a first point of differentiation between UFH, LMWHs, and fondaparinux is their chemical composition and mechanisms of action.

UFH is a heterogeneous mixture of highly sulfated mucopolysaccharides of varying lengths with molecular weights ranging from 3,000 to 30,000 Da (mean 15,000 Da) that has been used as an anticoagulant since the 1950s. However, its mechanism of action was not described until the 1970s. Only about one-third of the heparin molecules contain the pentasaccharide binding sequence required for its anticoagulant effect. By binding to AT, heparin induces a conformational change that facilitates the binding of AT to thrombin and catalyzes the inhibitory action of AT on both thrombin and Factor Xa. Heparin subsequently dissociates from the thrombin/AT complex and can be reutilized [14]. The other high-molecular-weight chains with low anticoagulant properties exhibit protein- and cell-binding effects and contribute to bleeding and other side-effects of UFH, such as thrombocytopenia and osteopenia [14].

In the 1980s, the LMWHs were produced using physical, chemical, or enzymatic depolymerization processes. Each LMWH consists of a heterogeneous mixture of polysaccharide chains with molecular weights ranging from 2,000 to 9,000 Da (mean 4,000-5,500 Da, depending on the LMWH). As with UFH, the LMWHs bind to AT and catalyze the inhibition of Factor Xa but, as the polysaccharide chains are much smaller than those in UFH, the majority of LMWH molecules are too short to bridge AT to thrombin. Accordingly, LMWHs have a much greater inhibitory effect on Factor Xa than on thrombin. Whereas UFH has an anti-Factor Xa to anti-Factor IIa ratio of 1:1, the corresponding ratio for the LMWHs ranges from 2:1 to 14:1. The shorter mucopolysaccharide chains also mean that the LMWHs have reduced cell and plasma protein binding compared to UFH. This translates clinically into a more predictable dose-response effect and a lower potential for LMWHs to induce effects such as heparin-induced thrombocytopenia. Each of the LMWH preparations has a different mixture of polysaccharide units due to the specific depolymerization process used in their manufacture, i.e. enoxaparin by alkaline depolymerization of heparin benzyl ester, dalteparin by nitrous acid depolymerization, and tinzaparin by enzymatic degradation using heparinase. This variation among the LMWHs gives rise to the different pharmacokinetic and pharmacodynamic profiles of the different LMWH agents [14].

Fondaparinux is a synthetic pentasaccharide with a molecular weight of 1,728 Da containing an AT-binding domain modified from that found in UFH and LMWHs to increase its affinity for AT. Fondaparinux cannot inhibit thrombin and its anticoagulant activity is entirely dependent on its ability to selectively inhibit Factor Xa [15]. Fondaparinux can be differentiated further from the polypharmacologic effects of UFH and LMWHs, as it does not release tissue factor pathway inhibitor [16].

### Use of parenteral anticoagulants throughout the continuum of VTE care

Translation of the differences in chemical profiles of the parenteral anticoagulants to their clinical profile is not straightforward. Parenteral anticoagulants have different FDA-approved indications for VTE, and there are differences in the supporting clinical evidence. Table 1 shows that, of the LMWHs, enoxaparin is licensed for the broadest range of VTE indications, encompassing both the prevention and treatment of VTE, based on the strength and depth of supporting clinical evidence. Tinzaparin is licensed for the treatment of VTE but not for VTE prophylaxis in any patient group, while dalteparin is licensed for VTE prevention but not for VTE treatment other than long-term treatment in cancer patients. Fondaparinux has received FDA approval for VTE prevention after orthopedic and abdominal surgery and for treatment of VTE (Table 1).

**Table 1:** Food and Drug Administration-approved indications of parenteral anticoagulants available for the treatment and prevention of venous thromboembolism in the US

Indication	Enoxaparin	Dalteparin	Tinzaparin	Fondaparinux
Prophylaxis				
Hip replacement surgery	Yes	Yes	No	Yes
Knee replacement surgery	Yes	No	No	Yes
Hip fracture surgery	No	No	No	Yes
Abdominal surgery	Yes	Yes	No	Yes
Acutely ill medical patients	Yes	Yes	No	No
Treatment	Yes	No	Yes	Yes
	- Inpatient DVT with/without PE - Outpatient DVT without PE		- Inpatient DVT with/without PE	- DVT - PE when initial therapy is administered in the hospital
Secondary prophylaxis/extended treatment in cancer patients	No	Yes	No	No

DVT, deep-vein thrombosis; PE, pulmonary embolism.

#### In-hospital VTE prophylaxis

Meta-analyses of randomized controlled clinical studies have shown that the LMWHs are at least as safe and effective as UFH in preventing VTE in both medical and surgical inpatients [16,17]. In general surgery, a meta-analysis of 51 studies involving more than 48,000 patients reported that prophylaxis with LMWHs was associated with a significant reduction in the incidence of clinical VTE compared to prophylaxis with UFH (p = 0.049) with a similar rate of major bleeding complications (p = 0.16) [18]. In hospitalized medical patients, a meta-analysis of nine trials including data from 4,669 patients demonstrated that the LMWHs and UFH were both similarly effective in reducing the incidence of VTE, but LMWHs were associated with 52% fewer bleeding complications than with UFH (p = 0.049) [17].

Meta-analyses are valuable to confirm the favorable risk-to-benefit profile associated with LMWH prophylaxis but they include data from trials of various LMWH preparations and cannot be used to support prescribing decisions for an individual LMWH. For this reason, the efficacy and safety data from the clinical studies for each LMWH need to be reviewed. Table 2 summarizes the key published data in the prevention of VTE for each of the LMWHs currently available in the USA and fondaparinux [13,19-40].

**Table 2:** Clinical evidence from randomized controlled trials for the efficacy and safety of parenteral anticoagulants in the prophylaxis of VTE

Indication/agent	**Ref**.	N	Dose	Comparator	VTE, %	Major bleeding, %
Hip replacement surgery						
Enoxaparin	[19]	100	30 mg bid	Placebo	12 vs 42 (p = 0.0007)	2 vs 4
	[20]	665	30 mg bid	UFH bid	17.1 vs 19.0	3.3 vs 5.7
	[21]	607	40 mg once daily	UFH tid	15 vs 12	1 vs 6 (p = 0.014)
			30 mg bid		5 vs 12 (p = 0.03)	4 vs 6
	[22]	3,011	30 mg bid	Warfarin	0.3 vs 1.1* (p = 0.0083)	0.6 vs 0.3
Dalteparin	[23]	1,472	2,500 pre-op and post-op, 5,000 IU once daily	Warfarin	10.7 vs 24 (p < 0.001)	2.2 vs 0.4 (p = 0.01)
			2,500 post-op, 5,000 IU once daily		13.1 vs 24 (p < 0.001)	0.8 vs 0.4
Tinzaparin	[24]	440	4,500 IU	Enoxaparin 40 mg once daily	21.7 vs 20.1	0.9 vs 1.8
Fondaparinux	[25]	2,309	2.5 mg once daily	Enoxaparin 40 mg once daily	4 vs 9 (p < 0.0001)	4 vs 3
	[26]	1,584	2.5 mg once daily	Enoxaparin 30 mg bid	6 vs 8	2.0 vs 0.7
Knee replacement surgery						
Enoxaparin	[27]	670	30 mg bid	Warfarin	36.9 vs 51.7 (p = 0.003)	2.1 vs 1.8
	[28]	349	30 mg bid	Warfarin	25.4 vs 45.5 (p = 0.0001)	5.2 vs 2.3
Fondaparinux	[29]	724	2.5 mg bid	Enoxaparin 30 mg bid	12.5 vs 27.8 (p < 0.001)	2.1 vs 0.2 (p = 0.006)
Hip fracture surgery						
Fondaparinux	[30]	1,250	2.5 mg once daily	Enoxaparin 40 mg once daily	8.3 vs 19.1 (p < 0.001)	2.2 vs 2.3
Abdominal surgery						
Enoxaparin	ENOXACAN [31]	631	40 mg once daily	UFH tid	14.7 vs 18.2	4.1 vs 2.9
Dalteparin	[13]	3,809	2,500 IU	UFH bid	1.0 vs 1.1*	3.6 vs 4.8
	[32]	1,957^†^	5,000 IU	Dalteparin 2,500 IU	6.6 vs 12.7 (p < 0.001)	1.3 vs 0.3
Fondaparinux	[33]	2,048	2.5 mg once daily	Dalteparin 2,500 pre-op and post-op, 5,000 IU once daily	4.6 vs 6.1 (p = 0.144)	3.4 vs 2.4 (p = 0.122)
Acutely ill medical inpatients						
Enoxaparin	MEDENOX [34]	866	40 mg once daily	Placebo	5.5 vs 14.9 (p < 0.001)	3.4 vs 2.0
	[35]	959	40 mg once daily	UFH tid	0.2 vs 1.4*	0.4 vs 1.5
	[36]	451	40 mg once daily	UFH tid	8.4 vs 10.4 (p = 0.015 for equivalence)	0.3 vs 0.3
	[37]	212	40 mg once daily	UFH 5,000 U tid	19.7 vs 34.7 (p = 0.044)	2.8 vs 1.9
	[38]	1,762	40 mg once daily	UFH 5,000 U bid	10 vs 18 (p = 0.0001)	1 vs 0 (p = 0.015)
Dalteparin	PREVENT [39]	3,706	5,000 IU	Placebo	2.77 vs 4.96 (p = 0.0015)	0.49 vs 0.16 (p = 0.15)
Fondaparinux	ARTEMIS [40]	849	2.5 mg once daily	Placebo	5.6 vs 10.5 (p = 0.029)	0.2 vs 0.2

*Incidence of symptomatic VTE diagnosed during hospitalization.

^†^Intent-to-treat population.

bid, twice daily; tid, three times daily; UFH, unfractionated heparin; VTE, venous thromboembolism.

Enoxaparin has been evaluated in multiple randomized controlled trials in different patient populations and against different comparators. In patients undergoing hip replacement surgery, enoxaparin 30 mg twice daily (bid) was associated with a lower incidence of symptomatic or asymptomatic VTE versus placebo (12% vs 42%; p = 0.0007) with low rates of major bleeding (2% and 4%, respectively) [19]. Several trials comparing enoxaparin 40 mg once daily or 30 mg bid with UFH bid or three times daily (tid) in this patient population demonstrated at least similar incidence rates of VTE and major bleeding [20,21]. In one study, enoxaparin 30 mg bid was associated with a lower rate of VTE (5% vs 12%; p = 0.03) and no increased bleeding (4% vs 6%) compared with UFH [21]. Compared with warfarin, enoxaparin 30 mg bid was associated with a lower incidence of symptomatic VTE during hospitalization (1.1% vs 0.3%, respectively; p = 0.0083) with major bleeding rates of 0.3% and 0.6% [22]. In trials in patients undergoing knee replacement surgery, there was a consistently lower incidence of VTE with enoxaparin 30 mg bid than with warfarin with no significant increase in the rate of major bleeding [27,28]. In patients undergoing abdominal or pelvic surgery for cancer, enoxaparin 40 mg once daily was as effective and safe as UFH administered tid [31]. In acutely ill medical patients, the incidence of symptomatic or non-symptomatic VTE was lower with enoxaparin 40 mg once daily than with placebo (5.5% vs 14.9%; p < 0.001) with no significant increase in major bleeding (3.4% vs 2.0%) [34]. Enoxaparin 40 mg once daily was as safe and effective as UFH 5000U tid [35,36]. In patients with acute ischemic stroke, the risk of symptomatic or asymptomatic VTE with enoxaparin 40 mg once daily was lower compared to that with UFH 5,000 U bid (10% vs 18%; p = 0.001), with a similar risk of symptomatic intracranial bleeding (1% vs 1%; p = 0.55) and a higher rate of major extracranial bleeding (1% vs 0; p = 0.015) [38] (Table 2).

Dalteparin has also been evaluated for the prevention of VTE. In patients undergoing hip replacement surgery, dalteparin 5,000 IU once daily was associated with a lower incidence of VTE compared with warfarin (11.9% vs 24.0%; p < 0.001), while major bleeding was higher with preoperative initiation of dalteparin (2.2% vs 0.4%; p = 0.01) but not postoperative initiation (0.8% vs 0.4%) [23]. In abdominal surgery, dalteparin 2,500 IU was as safe and effective as UFH bid [13]. In acutely ill medical patients, the incidence of symptomatic or asymptomatic VTE was lower with dalteparin 5,000 IU versus placebo (2.77% vs 4.96%; p = 0.0015) with no significant increase in major bleeding (0.49% vs 0.16%; p = 0.15) [39]. Few randomized controlled trials have evaluated the off-label use of tinzaparin in the prevention of VTE [24] (Table 2).

Trials with fondaparinux 2.5 mg once daily in hip replacement surgery demonstrated a lower incidence of VTE when compared with enoxaparin 40 mg once daily [25], but an equivalent incidence compared with enoxaparin 30 mg bid [26], while reporting no significant difference in the incidence of major bleeding. In knee replacement surgery, fondaparinux 2.5 mg bid was associated with a lower incidence of asymptomatic or symptomatic VTE compared with enoxaparin 30 mg bid (12.5% vs 27.8%; p < 0.001), at the expense of an increase in major bleeding complications (2.1% vs 0.2%; p = 0.006) [29]. In hip fracture surgery, fondaparinux 2.5 mg once daily was compared with enoxaparin 40 mg once daily, and showed a lower rate of VTE (8.3% vs 19.1%; p < 0.001) with no significant difference in major bleeding rates (2.2% vs 2.3%; p = 1.0) [30]. In a meta-analysis of 4 trials in major orthopedic surgery patients, the incidence of symptomatic or asymptomatic VTE with fondaparinux was lower compared with enoxaparin (6.8% vs 13.7%; p < 0.001) with an increase in major bleeding events (2.7% vs 1.7%; p = 0.008) [41]. Fondaparinux 2.5 mg once daily in patients undergoing abdominal surgery was associated with similar rates of VTE (4.6% vs 6.1%; p = 0.144) and major bleeding (3.4% vs 2.4%; p = 0.122) compared with dalteparin 5,000 IU started preoperatively at a dose of 2,500 IU [33]. In acutely ill medical patients at risk of VTE, fondaparinux was associated with a lower incidence of asymptomatic or symptomatic VTE compared with placebo (5.6% vs 10.5%; p = 0.029) and did not increase major bleeding (0.2% vs 0.2%) [40] (Table 2).

#### Extended-duration thromboprophylaxis

It has been reported that more than two-thirds of all symptomatic VTE events occur in the outpatient setting, predominantly in patients who have recently undergone surgery or been hospitalized for medical illness [3]. In fact, 47% and 76% of all clinical VTE events related to hip or knee replacement, respectively, occur post-discharge [42]. Currently, both the ACCP and IUA guidelines recommend prophylaxis extended beyond hospitalization to up to 35 days in specific patient groups, such as patients undergoing major elective orthopedic surgical procedures or major surgery for cancer [6,8].

The majority of the available clinical evidence for the use of LMWHs as extended-duration prophylaxis is with enoxaparin. In orthopedic surgery, three randomized, double-blind placebo-controlled trials have shown that a lower incidence of VTE can be achieved by extending the duration of prophylaxis with enoxaparin to 4 weeks postoperatively, without an increasing in the number of bleeding complications or other adverse events [43-45]. Similarly, a double-blind randomized controlled study in patients undergoing major surgery for abdominal or pelvic cancer has shown that 4 weeks of prophylaxis with enoxaparin 40 mg once daily is significantly more effective in reducing VTE than 1 week of enoxaparin prophylaxis (4.8% vs 12.0%; p = 0.02) with no increase in major bleeding (0.8% vs 0.4%; p > 0.99); this benefit was sustained during a 3-month follow-up period [46].

Dalteparin has been evaluated for extended-duration prophylaxis in surgical patients; two double-blind placebo-controlled studies have shown a lower incidence of late VTE with dalteparin in patients undergoing total hip replacement surgery without increasing major bleeding [47,48]. A small study (n = 176) on extended-duration prophylaxis with tinzaparin after general surgery failed to demonstrate a significant difference between 1 and 4 weeks of prophylaxis (VTE incidence 5.2% vs 10%; p = 0.49) and reported similar rates of bleeding (2/58 vs 3/60) [49].

Fondaparinux has been shown to be effective as extended-duration prophylaxis after major orthopedic surgery in two trials. The placebo-controlled PENTHIFRA study reported that in patients who had undergone hip fracture surgery extending the duration of prophylaxis with fondaparinux for 3 weeks was associated with a lower risk of symptomatic VTE (0.3% vs placebo 2.7%; p = 0.02) with an increase in major bleeding complications bordering significance (2.4% vs 0.6%; p = 0.06) [50]. Similarly, 3-5 weeks of extended-duration VTE prophylaxis with fondaparinux was shown to be effective and have a good safety profile after major lower limb surgery in patients with or without indwelling neuraxial or deep peripheral nerve catheters [51].

#### Treatment of VTE

Current ACCP guidelines recommend LMWH, UFH, or fondaparinux for the short-term treatment of objectively confirmed VTE [7]. Treatment with a LMWH given subcutaneously once daily or bid is recommended over UFH, on an outpatient basis if possible, and as an inpatient if necessary [7]. A number of clinical studies [52-57] and a meta-analysis [58] have demonstrated that the LMWHs are superior or non-inferior to UFH in preventing VTE recurrence (pooled relative risk [RR] 0.85, 95% confidence interval [CI]: 0.65-1.12; N = 4,447) and the incidence of bleeding events (pooled RR 0.63, 95% CI: 0.37-1.05; N = 4,447), and superior to UFH in terms of improved survival (pooled RR 0.76, 95% CI: 0.59-0.98; N = 4,033) [58]. The key clinical studies of each of the LMWHs in the treatment of VTE [52-57] are shown in Table 3[52-57,59-61].

**Table 3:** Evidence from clinical trials of the efficacy and safety of parenteral anticoagulants in the treatment of VTE

Indication/agent	**Ref**.	N	Dose	Patients	Comparator	Recurrent VTE, %	Major bleeding, %
Inpatient VTE treatment							
Enoxaparin	[52]	900	1.5 mg/kg once daily	DVT with/without PE	IV UFH	4.4 vs 4.1*	1.7 vs 2.1*
			1.0 mg/kg bid			2.9 vs 4.1*	1.3 vs 2.1*
Dalteparin	[53]	204	200 IU/kg once daily	DVT	IV UFH	5.0 vs 2.9	0 vs 0
	[54]	253	200 IU/kg once daily	DVT	IV UFH	3.6 vs 1.7	0 vs 1.5
Tinzaparin	THESEE [55]	612	175 IU/kg once daily	PE	IV UFH	1.6 vs 1.9*	2.0 vs 2.6*
Fondaparinux	MATISSE [59]	2,213	5 mg (body weight <50 kg), 7.5 mg (50-100 kg), or 10 mg (>100 kg)	PE	IV UFH	3.8 vs 5.0*	2.0 vs 2.4*
	[60]	2,205	5 mg (body weight <50 kg), 7.5 mg (50-100 kg), or 10 mg (>100 kg)	DVT	Enoxaparin 1.0 mg/kg bid	3.9 vs 4.1*	2.6 vs 2.4*
Outpatient treatment							
Enoxaparin	[56]	500	1.0 mg/kg bid	DVT	IV UFH (inpatient)	5.3 vs 6.7	2.0 vs 1.2
	[61]	298	1.5 mg/kg once daily	DVT	IV UFH (inpatient)	2.7 vs 8.8 (p = 0.026)	0 vs 2.0
Tinzaparin	[57]	505	175 IU/kg once daily	DVT and/or PE	Dalteparin 200 IU/kg once daily	3.9 vs 3.6	2.0 vs 0.8

*Non-inferior.

bid, twice daily; DVT, deep-vein thrombosis; IV, intravenous; PE, pulmonary embolism; UFH, unfractionated heparin; VTE, venous thromboembolism.

Enoxaparin is the only LMWH currently licensed in the US for outpatient treatment of acute DVT (without PE) in conjunction with oral warfarin (Table 1). A meta-analysis of studies of enoxaparin outpatient treatment including more than 1,500 patients with DVT, with or without PE, reported that enoxaparin is as effective in terms of VTE recurrence, major bleeding, and death as inpatient treatment with UFH [62]. The presence of symptomatic PE does not appear to affect the outcomes of DVT treatment with enoxaparin [62]. Furthermore, enoxaparin continued beyond the initial treatment of DVT offers benefits; an analysis of outpatient treatment of VTE with enoxaparin found that outpatient treatment with a combination of enoxaparin and warfarin was associated with 26% (p < 0.05) fewer hospital readmissions for recurrent DVT or PE compared to patients treated with warfarin alone. This was associated with an overall saving of USD 1,151 per patient in total DVT-treatment-related costs over warfarin monotherapy [63].

Although clinical data are available for dalteparin in the treatment of VTE [53], it is not licensed by the FDA for VTE treatment other than long-term treatment in cancer patients (Table 1). The CLOT trial reported that long-term prophylaxis with dalteparin in patients with cancer and DVT was associated with a significantly lower incidence of VTE recurrence compared to oral anticoagulant therapy with no increase in bleeding complications [64]. Based on the findings of the CLOT trial [64], dalteparin is licensed for extended-duration prophylaxis in patients with cancer as a regimen of anti-Factor Xa 200 IU/kg once daily for 1 month followed by anti-Factor-Xa 150 IU/kg for an additional 5 months.

Tinzaparin is licensed for the inpatient treatment of DVT with or without PE [51] (Table 1). A study comparing tinzaparin 175 IU once daily with dalteparin 200 IU once daily for outpatient treatment of DVT and/or PE reported no significant difference in the incidence of recurrent VTE or major bleeding between the two LMWHs [57].

Fondaparinux is licensed for the outpatient treatment of DVT, and of PE if initial treatment takes place in the hospital (Table 1). Non-inferiority for VTE recurrence and major bleeding was demonstrated compared to UFH in the treatment of PE [59] and compared to enoxaparin in the treatment of DVT [60].

## Differentiation between branded and biosimilar LMWHs

Biosimilar versions of enoxaparin have been developed and being marketed as generic equivalents and clinically used in India and South America, and initial regulatory approval of a biosimilar formulation of enoxaparin was granted in Canada [65]. In July 2010, the US FDA approved a generic version of enoxaparin [Sandoz, a division of Novartis group] utilizing the generic drug pathway. On the other hand, the EMEA has considered these agents to be biosimilar. Furthermore, the EMEA stated that biosimilar versions of LMWHs should be considered biological medicinal products and may not be submitted for approval as generic medicinal products. In a concept guideline manuscript, the EMEA considers the heterogeneity of LMWH to be very high, and recommends clinical trials to demonstrate equivalence of biosimilar LMWHs [11]. Thus there is a regulatory discordance between the EMEA and US FDA.

As the biosimilar LMWHs have only recently been produced there is limited clinical data available with which to compare the biosimilar and branded versions. The available data from pharmacological *in vitro *or preclinical studies using anticoagulation profiles and neutralization with protamine sulfate as their primary outcomes differentiate branded from biosimilar LMWHs. Biosimilar and branded versions of enoxaparin [66,67] and dalteparin [67] differ in their responses to the inhibitor protamine sulfate, the composition of their oligosaccharide chains [66,67], their affinity for AT [67], and their immunogenic potential [68]. The differences in assay-responses become more pronounced at higher concentrations: for example, at prophylactic doses the anticoagulant levels of the branded and biosimilar enoxaparin appear similar but at treatment doses enoxaparin exhibits significantly greater anticoagulant effects [66]. Similar dose-dependent variations have been reported in the response to neutralization with protamine sulfate [67].

## Anticoagulants in development

(Ultra-)LMWHs are currently being developed for different indications, amongst others bemiparin (Rovi, Madrid, Spain) and semuloparin (sonofi-aventis, Paris, France). Due to the clinical and practical limitations of warfarin, new oral anticoagulants to replace warfarin have also been sought. Oral vitamin K antagonists (warfarin) are used for long-term anticoagulation [6,7]. The first of the new oral anticoagulants was the oral direct thrombin inhibitor ximelagatran. However, this drug was withdrawn from use soon after it was licensed due to evidence of its potential hepatotoxicity. Two second-generation direct oral Factor Xa inhibitors--rivaroxaban, which has been approved in the EU and Canada, and apixaban--are currently in the final stages of clinical development. Based on the ximelagatran experience, clinical vigilance is required to determine the long-term safety of these agents. Dabigatran is another novel oral anticoagulant which has received recent approval in some European countries, Canada and recently in US; dabigatran is a direct thrombin inhibitor, administered orally as a double prodrug formulation (dabigatran etexilate). However, while this drug is approved in EMEA for post orthopedic surgical thromboprophylaxis, in the US, it is approved for stroke prevention in atrial fibrillation. Several other oral anticoagulants such as edoxaban, betrixiban are also in clinical development. A parenteral anti Xa agent with strong anticoagulant activity namely Otamixaban (sonofi-aventis, Paris, France) is also in advanced clinical stages as a parenteral anticoagulant in percutaneous intervention.

New anticoagulants completing clinical development are not supported by the depth of clinical evidence that is available for the currently recommended anticoagulants in terms of clinical experience or the range of patient populations and indications. Many issues remain to be elucidated, such as long-term safety, drug initiation, reversal of anticoagulation, and the appropriate bridging protocol from parenteral agents.

## Conclusions

The use of antithrombotic agents in VTE management spans a continuum ranging from VTE prevention using prophylaxis of appropriate duration for at-risk patients to timely treatment of VTE. Ideally, an antithrombotic drug should have clinically proven efficacy with a good safety profile in both the prevention and treatment of VTE for a wide range of patient types. Each of the LMWHs is a distinct drug, with unique clinical pharmacokinetics and pharmacodynamics, and LMWHs cannot be prescribed interchangeably. LMWHs should be prescribed in accordance with recommended dose regimens for each licensed clinical indication, based on the available clinical evidence for each agent. The newer parenteral anticoagulant, fondaparinux, is now also licensed for VTE prophylaxis in surgical patients and the treatment of acute DVT; clinical experience with this anticoagulant is expanding. In contrast to the LMWHs, fondaparinux does not exhibit polypharmacologic action although thrombosis is a polypathologic process. Generic versions of the branded LMWHs are now available for clinical use. However, because of the intra-class heterogeneity of LMWHs, each LMWH needs to be supported by clinical evidence of its efficacy and safety profile. Of the currently available antithrombotics, the LMWH enoxaparin offers the most extensive clinical experience and the widest range of clinical indications including surgical and medical thromboprophylaxis, and inpatient and outpatient treatment of VTE. Besides the LMWHs, several ultra-LMWHs are currently being developed for different indications. Therefore, this class of drug will continue to have a major impact in the future management of thrombosis. The newly developed oral antithrombin and anti-Xa agents are mono-therapeutic and will require clinical validation in specific indications.

## Abbreviations

**ACCP**: American College of Chest Physicians; **AT**: antithrombin; **bid**: twice daily; **CI**: confidence interval; **DVT**: deep-vein thrombosis; **EMEA**: European Medicines Agency; **FDA**: Food and Drug Administration; **IUA**: International Union of Angiology; **LMWH**: low-molecular-weight heparin; **PE**: pulmonary embolism; **RR**: relative risk; **tid**: three times daily; **UFH**: unfractionated heparin; **VTE**: venous thromboembolism.

## Competing interests

Professor Fareed has received research grants from Bayer Pharma, Germany, and Gentium, Italy, and has served as a symposium speaker for sanofi-aventis, France, and King Pharma, USA. Dr Adiguzel and Dr. Thethi report no conflict of interest.

## Authors' contributions

JF created the concept for the manuscript, drafted the manuscript, and approved the final manuscript. CA and IT critically revised the manuscript for important intellectual content and approved the final manuscript.
